# Mechanical Compression by Simulating Orthodontic Tooth Movement in an In Vitro Model Modulates Phosphorylation of AKT and MAPKs via TLR4 in Human Periodontal Ligament Cells

**DOI:** 10.3390/ijms23158062

**Published:** 2022-07-22

**Authors:** Charlotte E. Roth, Rogerio B. Craveiro, Christian Niederau, Hanna Malyaran, Sabine Neuss, Joachim Jankowski, Michael Wolf

**Affiliations:** 1 Department of Orthodontics, Dental Clinic, University of Aachen, 52074 Aachen, Germany; croth@ukaachen.de (C.E.R.); cniederau@ukaachen.de (C.N.); michwolf@ukaachen.de (M.W.); 2 Helmholtz Institute for Biomedical Engineering, BioInterface Group, RWTH Aachen University, 52056 Aachen, Germany; hmalyaran@ukaachen.de (H.M.); sneuss-stein@ukaachen.de (S.N.); 3 Institute of Pathology, RWTH Aachen University, 52056 Aachen, Germany; 4 Institute for Molecular Cardiovascular Research (IMCAR), University Hospital RWTH Aachen, 52074 Aachen, Germany; jjankowski@ukaachen.de

**Keywords:** monoclonal antibody TLR4, compression force, MAPKs, AKT, human PDL, sterile inflammation

## Abstract

Mechanical compression simulating orthodontic tooth movement in in vitro models induces pro-inflammatory cytokine expression in periodontal ligament (PDL) cells. Our previous work shows that TLR4 is involved in this process. Here, primary PDL cells are isolated and characterized to better understand the cell signaling downstream of key molecules involved in the process of sterile inflammation via TLR4. The TLR4 monoclonal blocking antibody significantly reverses the upregulation of phospho-AKT, caused by compressive force, to levels comparable to controls by inhibition of TLR4. Phospho-ERK and phospho-p38 are also modulated in the short term via TLR4. Additionally, moderate compressive forces of 2 g/cm^2^, a gold standard for static compressive mechanical stimulation, are not able to induce translocation of Nf-kB and phospho-ERK into the nucleus. Accordingly, we demonstrated for the first time that TLR4 is also one of the triggers for signal transduction under compressive force. The TLR4, one of the pattern recognition receptors, is involved through its specific molecular structures on damaged cells during mechanical stress. Our findings provide the basis for further research on TLR4 in the modulation of sterile inflammation during orthodontic therapy and periodontal remodeling.

## 1. Introduction

Mechanical forces are used as a therapeutic tool enabling orthodontic tooth movement (OTM) to improve functional and aesthetical malocclusions [1,2]. The mechanical stimulation is transmitted from the teeth to the periodontal ligament (PDL) where it is translated into a biochemical reaction, that leads to the remodeling of the alveolar bone [2,3,4,5]. The tooth is anchored to the surrounding alveolar bone via the periodontium, which absorbs the various shocks associated with mastication and provides tooth stability by continuously remodeling its extracellular matrix, the periodontal ligament (PDL) [6]. It represents a specialized connective soft tissue with viscoelastic properties, mainly comprised of fibroblasts and extracellular matrix (ECM) with the following main functions: anchoring teeth by Sharpey’s fibers into the alveolar cavity [4], supply of tooth nutrients, homeostasis and tissue remodeling. Mechanical stimuli, initially directed to the ECM of the PDL, are transduced via mechanosensitive receptors, ion channels [7] and pattern recognition receptors (PRR) to biochemical responses. This leads to a local sterile inflammation with pro-inflammatory cytokines, e.g., interleukins (IL-6, IL-8) [8,9,10], vascular endothelial growth factor (VEGF) [11] and matrix metalloproteinases (MMP8 and 9) [10,12]. MMPs are a group of enzymes that in concert are responsible for the degradation of most extracellular matrix proteins during organogenesis, growth and normal tissue turnover and increase significantly in various pathological conditions [13] and serve to accurately predict the level of inflammation during OTM [14]. These factors trigger an immune response, resulting in migration, adhesion and differentiation of monocytes and osteoclasts [15,16,17]. The monocytes dislodge cellular debris and allow for the remodeling of periodontal architecture in the initial phase and in the later phase during the tooth movement [18,19]. This is followed by alveolar bone resorption in the compression area and bone formation in the tension area of the periodontal ligament.

As shown in in vitro and in vivo studies, Toll-like receptor 4 (TLR4), a type of pattern recognition receptors (PRR) family, is involved in osteoclastogenesis [20,21,22]. Furthermore, TLR4 has been considered an important player in the initiation and progression of diverse inflammatory diseases. Our previous study has shown that the TLR4 expression is significantly increased due to mechanical stimulation in periodontal cells [8]. TLR4 seems to play an important role in two common inflammation types of the periodontium. Firstly, the sterile inflammation resulting from trauma or injuries is characterized by the release of endogenous molecules termed damage-associated molecular patterns (DAMPs). Secondly, the infectious inflammation, triggered by conserved structural motifs found in microorganisms, called pathogen-associated molecular patterns (PAMPs), leads to periodontal diseases, e.g., periodontitis. Furthermore, TLR4 has been well characterized to be involved in the transduction of innate and adaptive host immune responses to microbial pathogens, such as lipopolysaccharides (LPS) [23]. Nowadays, many DAMPs are reported as TLR4 ligands, such as High Mobility Group Box 1 (HMGB1) which is described in PDL fibroblasts during OTM [8,24,25]. Thus, HMGB1 can be defined as an accessory regulator for bone remodeling during OTM. HMGB1 is a damage protein that is released in the extracellular medium through traumatic events [26,27]. However, the effect of mechanical cell stress in human PDL cells depends on variables such as force magnitude, duration and constancy [25,27]. These findings underline the need for further investigation of the signal transduction cascades involved in the mediation of mechanical cell stress into host immune responses.

We hypothesized that the expression of TLR4 in human PDL cells may be involved in the initial inflammation process in orthodontic tooth movement. In this study, we want to evaluate the role of TLR4 in human periodontal ligament cells in an in vitro compression model for OTM and TLR4 signaling during sterile inflammation. To this end, we investigated the modulation of TLR4 under mechanical stress and its involvement in key downstream molecules such as AKT, also known as protein kinase B, and the highly conserved mitogen-activated protein kinase (MAPK) family, with its three important members (extracellular signal-regulated kinase (ERK), p38 and c-Jun N-terminal kinase (JNK)). The TLR4 modulation regulates the cells by transducing extracellular into cellular responses and enables a better understanding of the molecular mechanisms underlining sterile periodontal remodeling.

## 2. Results

### 2.1. Characterization of Primary Human PDL Cells

**Figure 1:**
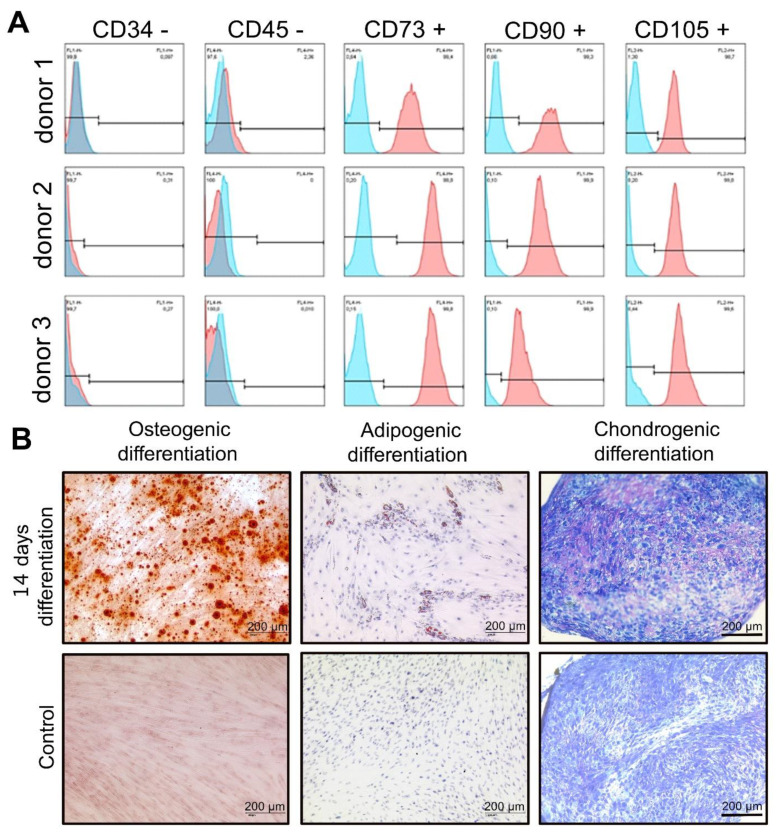
**Characterization of primary human PDL cells.** (**A**) hPDL cells were first characterized by flow cytometry analysis of cell surface markers. The expression pattern for all donors is CD34−, CD45−, CD73+, CD90+ and CD105+ (blue color: isotype control, red color: surface marker) and (**B**) hPDLs differentiation towards adipocytes, chondrocytes and osteoblasts was performed. hPDL cells were grown in osteogenic, adipogenic and chondrogenic differentiation media for 14 days and then stained with alizarin red, Oil red O and Toluidin blue, respectively. The red color represented mineralized calcium depositions after PDL cells differentiated into osteoblast-like cells. A large number of orange lipid vacuoles were seen in PDL cells after culture in AIM and staining with Oil red O. Staining with Toluidine blue revealed excessive production of extracellular matrix components and proteoglycans during the culture process with CIM (scale: 200 μm).

At first, three isolated PDL cells from the upper jaw were evaluated for stem cell characteristics via surface characterization according to the Guidelines of the International Society for Stem Cell Research. The percentages of specific stem cell markers were analyzed by flow cytometry. All donors express stem cell markers (CD34−, CD45−, CD73+, CD90+ and CD105+), sharing in addition morphological and phenotypical features specific for multipotent adult mesenchymal stem cells (MSCs). To demonstrate the multipotency of hPDLs, when cultured under the appropriate conditions, the mesodermal differentiation towards adipocytes, osteoblasts and chondrocytes was induced in three separate donors. The adipogenic induction results in the formation of lipid droplets, which were visualized by Oil red O staining, on the culture plate. The first lipid droplets were observed after 14 days. hPDLs, which were cultured in standard media showed no formation of lipid vacuole formation during the differentiation process. Chondrocytes produce large amounts of extracellular matrix composed of collagen, proteoglycan, and elastin. The differentiation towards chondrocytes was induced with CIM. Staining with Toluidine blue revealed excessive production of extracellular matrix components and proteoglycans during the culture process with CIM. In comparison with the control, the staining was darker and hyaline structures appeared more organized. Osteogenic differentiation was induced with OIM. hPDLs were seeded at a density of 31.000 cells/cm^2^ and stained with Alizarin red after 14 days in culture. Alizarin red stains calcium depositions that are characteristic of the osteogenic cell fate. Dark red formations represent the calcium depositions. Over the whole culture period, no red staining was observed in the control group (Figure 1).

**Figure 2:**
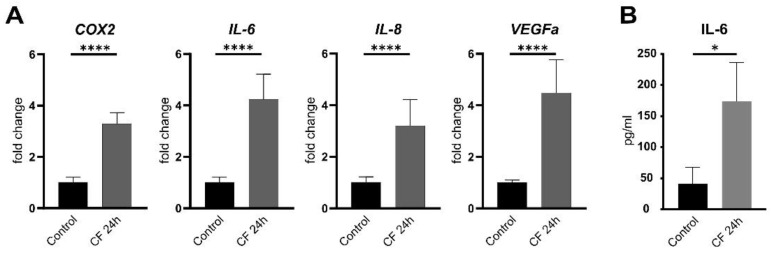
**Inflammatory markers are upregulated by compressive forces in hPDL after 24 h.** To analyze the regulation of inflammatory cytokines under compressive forces, the expression of different markers was analyzed on mRNA level (**A**) and protein levels (**B**) after mechanical stimulation. The pro-inflammatory cytokines interleukin-6 (IL-6), interleukin-8 (IL-8) and cyclooxygenase-2 (Cox2) are significantly upregulated. The vascular endothelial growth factor A (VEGFA), a mediator of inflammation and angiogenesis, is also significantly upregulated. The data represent two independent experiments in triplicates; normalization by ddC_t_ method to RPL22 and control 100% statistical data were tested for normal distribution by Shapiro–Wilk test and a *t*-test was performed. Statistically significant differences are marked by an asterisk (* *p* < 0.05, **** *p* < 0.0001).

Furthermore, as shown in previous studies, PDL cells are characterized by their ability to express inflammatory markers under mechanical compression [9,11]. Here, we also observed the upregulation of inflammatory cytokines under compressive force. The pro-inflammatory cytokines interleukin-6 (IL-6), interleukin-8 (IL-8) and cyclooxygenase-2 (Cox2) and the vascular endothelial growth factor A (VEGFA), a mediator of inflammation and angiogenesis, are significantly upregulated (Figure 2A,B).

### 2.2. Changes in the TLR4 Production under Compressive Forces

**Figure 3:**
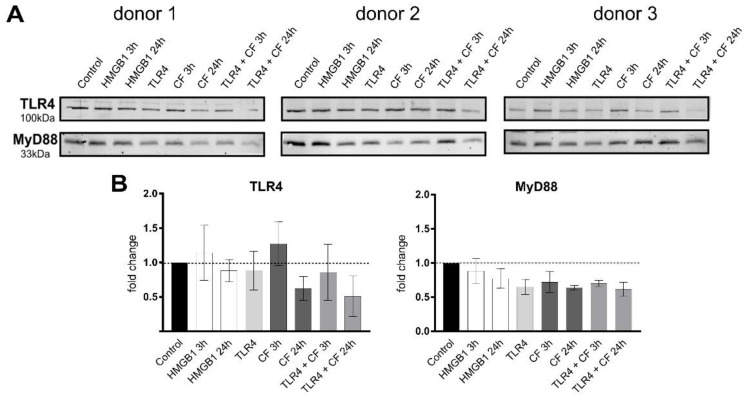
**Compressive forces and TLR4 blocking antibody modulate TLR4 production and MyD88 in primary PDL cells.** (**A**) Protein levels of TLR4 and MyD88 were determined by Western blot in different conditions: HMGB1 (100 ng/mL), TLR4 blocking antibody (5 µg/mL) (TLR4: TLR4 blocking antibody) and compressive force (CF) 2 g/cm^2^ for 3 and 24 h. Three different donors showed similar patterns with reduction of TLR4 antibody. TLR4 production was upregulated under 3 h compression forces. MyD88 production was reduced in all conditions. (**B**) Quantification of three donors, normalized to the control with stain-free technology. Data were tested for normal distribution by Shapiro–Wilk test. Afterward, a one-way analysis of variance (ANOVA) followed by Tukeys’ post hoc test was performed.

To investigate the effect of compressive forces on the TLR4 protein production, the ligand HMGB1 (100 ng/mL) was used as a stimulator, and TLR4 blocking antibody (5 µg/mL) as an inhibitor for the TLR4 downstream signaling. The TLR4 protein level was clearly upregulated after a short-term culture (3 h) of mechanical stimulation and downregulated after a long-term culture (24 h) (Figure 3). The same pattern was observed for all three donors (Figure 3). TLR4 block antibody alone did not affect the basal TLR4 production, but in combination with 3 h and 24 h compressive force, it was downregulated. The activation of TLR4 with HMGB1 resulted in a slightly higher production after 3 h without having an effect after 24 h. MyD88, a downstream adapter molecule, that plays a pivotal role in immune activation through TLRs, showed just a slight decrease for all conditions, except for a significant downregulation by compressive forces for 24 h (Figure 3).

### 2.3. Phospho-AKT Was Upregulated by Compressive Force

**Figure 4:**
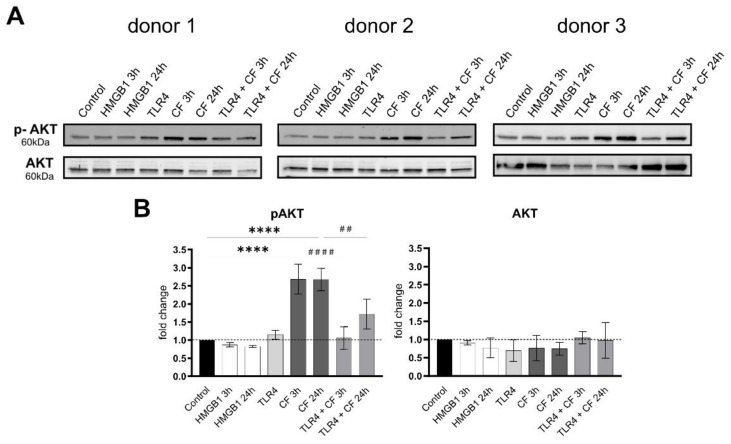
**Compressive forces upregulate phosphor AKT in primary hPDL cells.** (**A**) Protein production levels and phospho-AKT were determined by Western blot in different conditions: HMGB1 (100 ng/mL), TLR4 blocking antibody (5 µg/mL) (TLR4: TLR4 blocking antibody) and compressive force (CF) 2 g/cm^2^ at 3 and 24 h. Three different donors showed similar patterns. Under all conditions, AKT showed no differences, but the phosphorylated AKT was significantly upregulated with compressive forces (CF) for 3 h and 24 h. Compressive forces with additional blocking TLR4 monoclonal antibody led to significant downregulation comparable to the control. (**B**) Quantification of three donors, normalized to the control with stain-free technology. CF conditions without TLR4 blocking antibody showed a significant upregulation. Data were tested for normal distribution by Shapiro–Wilk test. Afterward, a one-way analysis of variance (ANOVA) followed by Tukeys’ post hoc test was performed. Statistically significant differences to control are marked by asterisks (**** *p* < 0.0001) and hashtag show significant differences between CF and TLR4 +CF (## *p* < 0.01; #### *p* < 0.0001).

To investigate downstream signaling of TLR4, AKT and its phosphorylation status was analyzed by Western blots under the same conditions. Under all conditions, AKT showed no differences, while the phosphorylated AKT was significantly upregulated with compressive forces (CF) for 3 h and 24 h. In particular, compressive forces with additional blocking TLR4 monoclonal antibody led to a significant downregulation comparable to the control (Figure 4).

### 2.4. Phospho-ERK and Phospho-p38 Were Significantly Upregulated under Compressive Force

**Figure 5:**
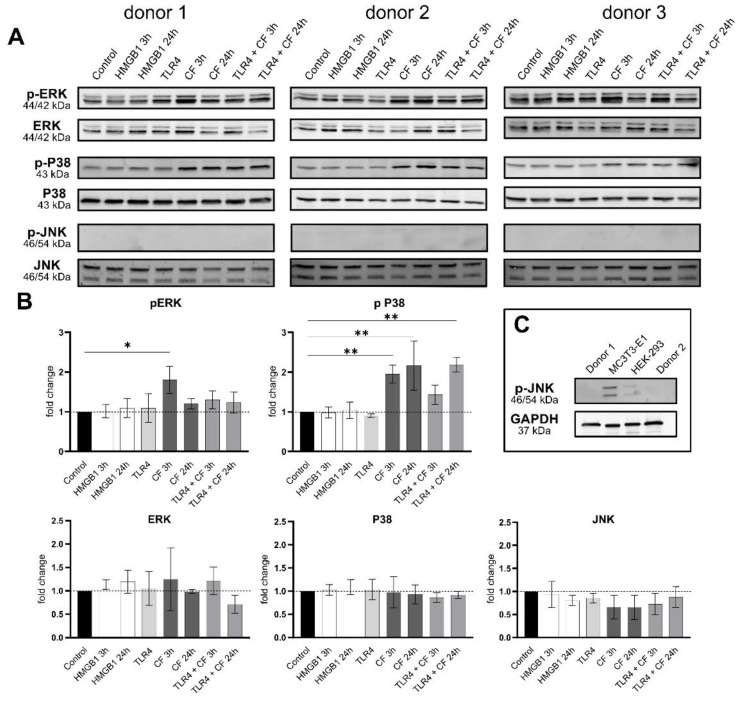
**Compressive forces upregulate pERK and p P38 in primary hPDL cells.** (**A**) Protein levels and phospho-MAPK (ERK, p38 and JNK) were determined by Western blot in different conditions: HMGB1 (100 ng/mL), TLR4 blocking antibody (5 µg/mL) (TLR4: TLR4 blocking antibody) and compressive force 2 g/cm^2^ at 3 and 24 h. Three different donors showed similar patterns. Under all conditions, ERK, p38 and JNK showed no differences, but the phosphorylated ERK and p38 were significantly upregulated with compressive forces (CF) as follows: ERK for 3 h and p38 for 3 h and 24 h. (**B**) Quantification of three donors, normalized to the control with stain-free technology. CF conditions without TLR4 blocking antibody showed a significant upregulation for phospho-ERK and phospho-p38. (**C**) HEK-293 and MC3T3 cells were used as a positive control for phospho-JNK antibody. Data were tested for normal distribution by Shapiro–Wilk test. Afterward, a one-way analysis of variance (ANOVA) followed by Tukeys’ post hoc test was performed. Statistically significant differences to control are marked by asterisks (* *p* < 0.05; ** *p* < 0.01).

Further, the phosphorylation status and production of MAP-Kinase (ERK, p38 and JNK) were investigated by Western blots. The MAPK did not change in all conditions. However, the phosphorylation of ERK (3 h) and p38 (3 and 24 h) was significantly upregulated under CF (Figure 5). The phosphorylation of ERK and p38 under 3 h compressive forces with additional blocking TLR4 monoclonal antibody was reduced. The phosphorylated form of JNK was not detected for PDL cells in any condition. The validation of antibody phosphorylation of JNK, however, was confirmed by a positive control (Figure 5C).

### 2.5. Moderate Compressive Forces on PDL Cells Is Not Able to Translocate NF-kB and ERK to the Nucleus

**Figure 6:**
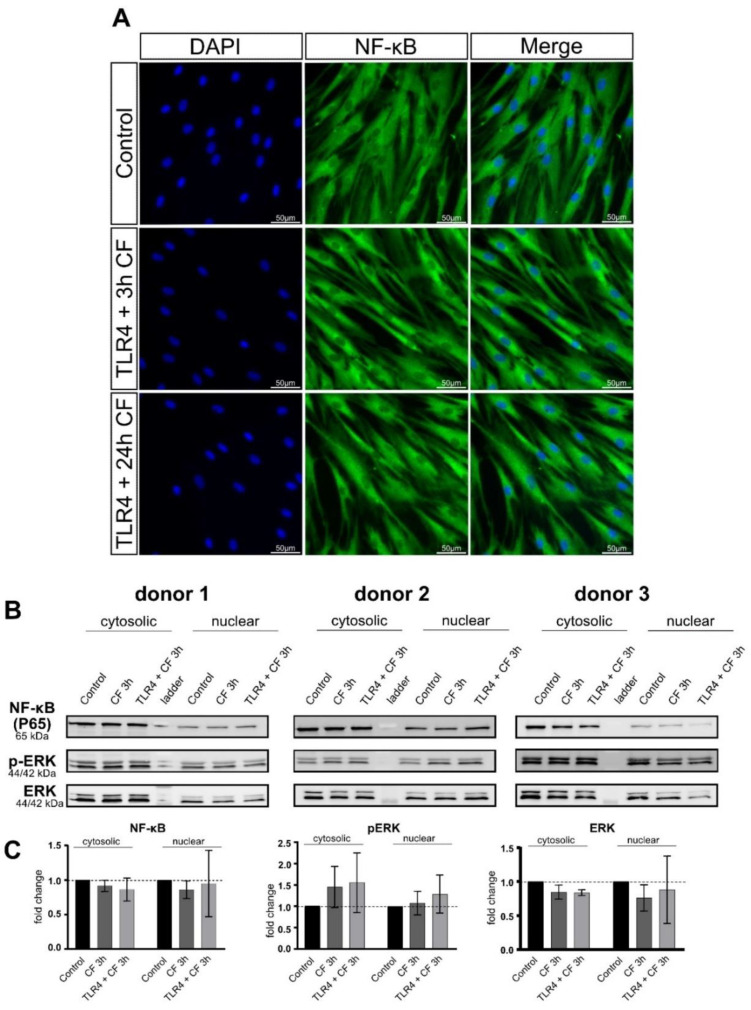
**Fluorescence images of CF and TLR4 blocking antibody on PDL cells.** (**A**) PDL cells treaded with and without TLR4 blocking antibody (5 µg/mL) and compressive force 2 g/cm^2^ for 24 h. NF-kB was stained green (Alexa 488), blue areas represent nuclei (DAPI); scalebar 50 µm, CF: compressive force NF-kB was not translocated to the nucleus in any condition. (**B**) Protein production of NF-kB, ERK and phospho-ERK were determined by Western blot. (**C**) Quantification of three donors, normalized to the control with stain-free technology. CF conditions without TLR4 antibody showed a significant upregulation for phospho-ERK and phospho-p38.

Inflammation is a process coordinated by the local secretion of adhesion molecules, chemotactic factors and cytokines [28]. The nuclear factor-kB (NF-kB), a transcription factor, is an important mediator for the activation of the IL-6 gene [29]. As shown in Figure 2, PDL cells are able to express inflammatory markers, in particular IL6, under mechanical compression. Based on this, we investigated, whether PDL cells under compressive force are able to translocate NF-kB or phospho-ERK to the nucleus and whether a TLR4 blocking antibody plays a role in this process. To study the translocation of NF-kB, fluorescence images and Western blot from cytoplasmic and nuclear fractions were performed. Fluorescence images for all conditions (control and 3 h compressive force with and without TLR4 blocking antibody) were not able to detect NF-kB translocation (Figure 6A). These results correspond to the data of the fractionated Western blot. NF-κB, as well as phosho-ERK did not lead to any changes in the cytosol and nuclear fraction (Figure 6B).

## 3. Discussion

The level of inflammatory markers in affected periodontal tissues during the orthodontic tooth movement (OTM) plays an important role as a viable diagnosis tool in monitoring the progression of the periodontium [14]. In our previous work, we reported that human PDL cells modify gene expression and protein production of pro-inflammatory cytokines via TLR4 signaling in an in vitro model, which represents the compressed periodontal ligament in the initial phase of OTM [8]. Hence in this study, we further explored the possible cell signaling downstream key molecules modulation via TLR4 caused by a sterile inflammation in three new isolated and characterized human PDL cells. To this end, we used the gold standard model for static compressive mechanical stimulation induced by static compression forces of 2 g/cm^2^ [9,11,30]. Some studies use this model with PDL cells to investigate mechanotransduction, i.e., the ability of a cell to actively sense, integrate, and convert mechanical stimuli into biochemical signals that result in intracellular changes, such as ion concentrations, activation of signaling pathways and transcriptional regulation [31,32,33]. Concerning mechanotransduction, this model can be used to explore, e.g., the mechanosensory protein complex and focal adhesion kinase (FAK) involved in mechanotransduction and subsequent YAP/TAZ translocation into the nucleus [34] or Wnt signaling responsive to mechanical loading [35], or mechanical strain modulating the amount of the matrix metalloproteinase MMP-13 [36]. Additionally, to this, we want to deepen the understanding of signal transduction cascades involved in mediating mechanical cell stress into host immune responses via TLR4 in a sterile inflammation by the release of damage-associated molecular patterns (DAMPs), resulting from injuries during OTM. TLR4 is very well investigated as a receptor involved in pathogenic models (LPS) PAMPs described in PDL cells [37,38]. Although TLR4 has been considered an important player in the initiation and progression of several inflammatory pathological conditions, we hypothesized that there might be a strong interplay between TLR4 and cell signaling in bacterial and sterile inflammation.

Differently from our previous work using a commercial primary cell—HPdLF [8], the presented data are based on self-isolated human PDL cells from the upper jaw of three different donors. Using only the samples from the upper jaw can help to avoid possible variability from primary PDL cells and support the level of reliability of our results. With newly isolated cells, we could confirm the gene expression and protein production under 24 h compressive force with results identical to our previous work. In our present study, it was observed that the TLR4 production increased after 3 h, and not after 24 h.

The PDL cells showed similar characteristics in the evaluation of the stem cell character as described in the literature for periodontal ligament stem cells [39]. In terms of morphology, surface epitopes and differentiation capacity towards adipocytes, osteoblasts and chondrocytes, PDLs behave comparably to MSCs. Moreover, the protein levels and phosphorylation status of different proteins analyzed in this study show very similar profiles in all Western blotting analyses for all three donors.

On the basis of our previous work about gene expression, similar results could be observed in this study, with TLR4 protein production increasing under compressive force and reducing with TLR4 monoclonal blocking antibody in a short-term culture. Moreover, the downstream signaling from TLR4 has clearly changed.

TLR4 can be activated by HMGB1 [40] and regulated under mechanical stress in PDL cells [41,42]. Furthermore, the HMGB1–TLR pathway is linked to the MyD88-mediated NF-κB pathway and activates downstream signaling pathways which in turn leads to the induction of innate immune responses by producing inflammatory cytokines and other mediators [43,44]. However, our results with HMGB1 show no significant changes in TLR4 and downstream molecules. The activation of TLR4 via HMGB1 might better work in a higher concentration or may be dependent on the complexes it forms with other molecules, immunostimulatory complexes, or with the binding of cytokines and other molecules [40,45,46,47]. HMGB1 binds to TLR4 with remarkably less affinity than LPS, and it activates gene expression of distinct signaling patterns after stimulation. Both HMGB1 and LPS significantly increase the nuclear translocation of NF-κB [40].

MyD88 used in this work as a downstream activation of TLR4 showed in all conditions a similar regulation pattern to the regulation of TLR4. After ligand binding, TLRs interact with adaptor proteins as myeloid differentiation primary response gene 88 (MyD88) or TIR domain-containing adaptor proteins and initiate signal transduction pathways that activate NF-κB or MAP kinases.

AKT is also known to be regulated by mechanical stimulation leading to activated FGF-2 production and to the involvement of FGF-2 in the PI3K/Akt or Rho pathway. In addition, AKT can be related to pro-inflammatory responses [48,49]. Our data confirmed that AKT is phosphorylated in response to a compressive force, being significantly upregulated with the compressive force for 3 h and 24 h. What is more, we could very clearly demonstrate that inhibition of TLR4 signaling via TLR4 monoclonal blocking antibody led to significant downregulation comparable to control.

In addition to AKT, mitogen-activated protein kinases (MAPKs), and other key molecules involved in signal transduction and localized downstream of TLR4, were evaluated. Recently, it has been published, that MAPKs are involved in mechanotransduction and resulting in inflammatory responses [50,51,52,53,54]. Our data also confirmed the modulation of phosphorylation of ERK and p38 by mechanical stimulation in human PDL cells. Additional blocking of TLR4 with a specific antibody reduced in a short-term culture, 3 h under compressive force, the phosphorylation of these MAPKs following mechanical stress. Interestingly, JNK does not seem to be regulated at all, which contrasts to several studies that showed regulation of phosphor-JNK in PDL cells due to mechanical compression. Of note, a phospho-JNK antibody recognized specifically the twice phosphorylated epitope at Thr183 and Tyr185. This indicates that if phospho-JNK binds only in one phospho residue, e.g., Tyr185, this antibody is not able to detect the phosphorylation of JNK.

LPS from Porphyromonas gingivalis leads to the activation of the TLR4/MyD88 complex, triggering the secretion of pro-inflammatory cytokine cascades as: IL-1α, IL-8, TNF-α and β and Eotaxin. Moreover, the upregulation of pERK/ERK signaling pathways and Nf-kB nuclear translocation was evident [55]. In our study, we could see that the activation of TLR4/MyD88 complex and modulation of phosphorylation of ERK and p38 triggering the secretion of pro-inflammatory cytokines work in a sterile inflammation during mechanical stress too. The strong correlation between these two common inflammation types of the periodontium triggered by PAMP or DAMP molecules might confirm that—as hypothesized [56]—the activation of pattern recognition receptor TLR4 shares certain biochemical actors that can regulate the inflammatory process in both types of inflammation. Based on this, the translocation of Nf-kB and phospho-ERK to the nucleus was analyzed by immunofluorescence as well as cytoplasmic and nuclear fractions by Western blotting. In a short and long compressive force time with and without TLR4 blocking antibody, no translocation of Nf-kB by immunofluorescence was observed. The same was observed in the nuclear and cytoplasmatic fractions of Nf-kB and phospho-ERK. Taking both results together, we could conclude that a moderate compressive force of 2 g/cm^2^ is not able to induce translocation of Nf-kB and phospho-ERK into the nucleus. However, despite this, inflammatory cytokines in gene expression, as well as protein levels of IL-6, were observed in our experiments. The activation of TLR4 through damage proteins during mechanical compression in this model may require less affinity compared with LPS. Another reason could be that the effect of mechanical cell stress on TLR production in human PDL cells is dependent on variables such as force magnitude, duration and constancy [25].

We could clearly demonstrate that in PDL cells under compressive forces, the inflammatory markers, as well as IL-6 protein production, are upregulated—as expected in line with other publications. Further, we could see that the use of TLR4 monoclonal blocking antibody significantly reverses the upregulation of phospho-AKT, caused by compressive force, to levels comparable to controls. The inhibition of TLR4 may indicate that this modulation is induced not only by mechanotransduction from mechanoreceptors as described by now. However, this study must be seen in the light of certain limitations since we could not describe or suggest a cell signaling mechanism independent from NF-kB or phosphor-ERK translocation for explaining the upregulation of inflammatory markers.

## 4. Materials and Methods

### 4.1. Reagents and Methods

Primary antibodies p38 MAPK (8690S), Phospho-p38 MAPK (9216S), p44/42 MAPK (Erk1/2) (3A7) (9107S), Phospho-p44/42MAPK (Erk1/2) (Thr202/Tyr204) (D13.14.4E) (4370S), SAPK/JNK (9252), Phospho-SAPK/JNK (Thr183/Tyr185) (G9) (9255S), Akt (pan) (40D4) (2920S), Phospho-Akt (Ser473) (D9E) (4060S) and MyD88 (D80F5) (4283) were purchased from Cell Signaling and TLR4 (MA5-16216) from Thermo Fisher Scientific, Waltham, MA, USA. Secondary antibodies StarBright Blue 700 (12004158) and StarBright Blue 520 (12005869) were purchased from Bio-Rad, Hercules, CA, USA. For flow cytometry APC, FITC and PE-labeled antibodies were purchased from eBiosciences, San Diego, CA, USA (FITC-labeled anti-human CD34 (11-0349-42), APC-labeled anti-human CD45 (17-0459-42), APC-labeled anti-human CD73 (17-0739-42), FITC-labeled anti-human CD90 (11-0909-42), PE-labeled anti-human CD105 (12-1057-42)). For Western blotting, RIPA buffer (Thermo Fisher Scientific, Waltham, MA, USA) complemented with cOmplete Tablets Mini and PhosStop (Roche, Basel, Switzerland), Neutralization monoclonal blocking antibody TLR-4 (HTA125) (14-9917-82) 5 μg/mL from Thermo Fisher Scientific, Recombinant Human HMGB1 Protein (1690-HMB) R&D Systems was used.

### 4.2. Primary Human Periodontal Ligament (hPDL) Cell Isolation

Primary human periodontal ligament (hPDL) cells were cultured from periodontal connective tissue, isolated from the middle root section of healthy human teeth. Only decay-free teeth from healthy donors, which needed to be extracted for medical reasons were used for human PDL cell isolation. Immediately after extraction, the teeth were transferred into an isolation medium of DMEM high-glucose (Gibco, Billings, MT, USA), 10% Fetal Bovine Serum (FBS), qualified heat-inactivated (Gibco), 50 mg/L ascorbic acid (Sigma, Saint Louis, MO, USA), antibiotic-antimycotic (Gibco) and stored at RT until isolation was started within 24 h after extraction. For isolation, residual tissue was mechanically scraped off and incubated in a solution of 3 mg/mL collagenase type I (Worthington Biochem, Freehold, NJ, USA) for 1 h at 37 °C. After incubation, cells were plated into 6-well plates. After the first splitting, the isolation medium was replaced by a culture medium of DMEM high-glucose (Gibco), 10 % FBS (Gibco), 50 mg/L ascorbic acid (Sigma), Penicillin/Streptomycin (Gibco). Human PDL cells (upper jaw, third molar, 28) from three different patients were used (1 male, 2 females, age: 19–22 years). Collection and usage of hPDL cells from discarded patient samples were approved by the ethics committee of the RWTH Aachen, Germany (approval number EK374/19), and all experiments were carried out in accordance with the relevant guidelines and regulations. Informed consent was obtained from all participants and/or their legal guardian/s. In this work, human PDL cells were used from passages two to five.

### 4.3. Characterization of hPDL Cells Flow Cytometry Analysis of Cell Surface Markers

To characterize the cells from the three different donors, the expression of specific stem cell markers was analyzed by flow cytometry. Briefly, 250,000 isolated hPDL cells were trypsinized and resuspended in FACS buffer (PBS + 0.1% FCS) and centrifuged for 5 min at 300× *g* at 4 °C. Afterwards, the cells were incubated for 30 min in the dark with primary PE/FITC/APC-coupled antibody CD34, CD45, CD73, CD90, and CD105 in a concentration of 0.5 µg, 0.06 µg, 0.125 µg and 1 µg per 250,000 cells, respectively. Cells were centrifuged for 5 min at 300× *g* and resuspended in 300 µL FACS buffer before measurement with BD FACSCalibur™ Flow Cytometer (BD Science, Franklin Lakes, NJ, USA). Analysis was performed using BD Cell Quest Pro Software from BD Science, USA.

### 4.4. PDL Cell Differentiation

Differentiation towards adipocytes: To induce the differentiation into an adipogenic phenotype, PDL cells were seeded in a density of 80.000 cells/cm² on TCPS incubated at 37 °C with 5% CO_2_ in a humidified atmosphere for 14 days. The medium was changed the next day to an adipogenic induction medium (AIM) containing DMEM high-glucose (Gibco, Darmstadt, Germany), 10% FCS (PAN-Biotech, Aidenbach, Germany) and the following supplements: 1 μM Dexamethasone, 0.2 μM Indomethacin, 0.5 mM IBMX, 0.01 mg/mL human Insulin (all Sigma Aldrich, Steinheim, DE, Germany) and 1% LGPS (80 U/mL Penicillin; 80 μg/mL Streptomycin; 1.6 mM L-Glutamine; Gibco, Germany). Medium change was performed two times a week, alternating with adipogenic induction medium (AIM) and adipogenic maintenance medium (AMM) containing DMEM high-glucose (Gibco, Germany), 10% FCS (PAN-Biotech, Germany), 0.01 mg/mL human Insulin (Sigma Aldrich, Germany) and 1% LGPS (80 U/mL Penicillin; 80 μg/mL Streptomycin; 1.6 mM L-Glutamine).

Oil red O Staining: To prove the success of adipogenic differentiation, Oil red O staining was performed. The staining solution was prepared by mixing 35 mL 0.2% (*w/v*) Oil red O powder (Sigma Aldrich, Darmstadt, Germany) in 100% ethanol (Merck, Darmstadt, Germany) with 10 mL 1 M NaOH (Merck, Germany) and filtrated. Cells were fixed in 50% ice-cold ethanol (4 °C) for 30 min and stained with Oil red O for 10 min. The supernatant was removed and cells were rinsed with 50% ethanol and aqua ad iniectabilia (B. Braun, Melsungen, Germany). Cell nuclei were stained blue by hemalum (Abcam, Cambridge, UK) and rinsed with tap water afterward.

Differentiation towards chondrocytes: Differentiation toward chondrocytes was induced with a chondrogenic induction medium (CIM) containing DMEM high-glucose (Gibco, Germany) and the following supplements: 100 nM Dexamethasone, 0.17 mM L-Ascorbic-Acid-2-phosphate, 100 μg/mL Sodium pyruvate, 4 μg/mL L-Proline (all Sigma Aldrich, Germany), 10 ng/mL TGF-β3 (R&D Systems, Wiesbaden, Germany), 1% LGPS (80 U/mL Penicillin; 80 μg/mL Streptomycin; 1.6 mM L-Glutamine; Gibco, Germany) and 5% ITS-Plus Premix (6.25 μg/mL Bovine Insulin, 6.25 μg/mL Transferrin, 6.25 μg/mL Selenium acid, 6.25 μg/mL Linoleic acid, 6.25 μg/mL BSA; Life Technologies, Darmstadt, Germany). Cells were seeded as pellet cultures in a density of 250,000 cells/0.5 mL and transferred to a 15 mL centrifuge tube, centrifuged for 7 min at 500 g and incubated at 37 °C with 5% CO2 in a humidified atmosphere for 14 days. The medium was changed from standard medium to CIM the next day after seeding and changed three times per week. The growth factor TGF-β3 was added freshly to the medium (0.5 μL/mL).

Toluidine blue staining: Cell pellets were fixed in 4% formalin (Morphisto, Karlsruhe, Germany) overnight at 4 °C and embedded in 3% agarose (Sigma Aldrich, Germany), dehydrated in an ascending ethanol series, treated with xylol and histoplast in a tissue processor. Pellets were embedded in paraffin and sectioned with a rotating microtome into slices of 2 μm thickness. Afterward, paraffin sections were incubated at 60 °C for 10 min. Paraffin residues were removed by placing the slides in xylene for 5–10 min and a descending alcohol series was performed (10 min each in 100%, 96%, 70% ethanol and aqua dest. (B. Braun, Germany)). Pellets were stained with toluidine blue (2 g toluidine blue powder (Sigma Aldrich, Germany) in acetate buffer (Merck, Germany), pH 4.66) for 2 min. Next, an ascending alcohol series was applied (96%, 100% ethanol and xylene for 1 min each). Finally, the samples were encapsulated in Vitro-Clud^®^ (R. Langenbrinck, Emmendingen, Germany) and investigated under a light microscope.

Differentiation towards osteoblasts: Induction towards osteoblasts was performed by osteogenic induction medium (OIM) containing DMEM low glucose (Gibco, Germany), 10% FCS (PAN-Biotech, Germany) and the following supplements: 100 nM Dexamethason, 10 mM Sodium-β-glycerophosphate, 0.05 mM L-Ascorbic-Acid-2-phosphate (all Sigma Aldrich, Germany) and 1% LGPS (80 U/mL Penicillin; 80 μg/mL Streptomycin; 1.6 mM L-Glutamine; Gibco, Germany). Cells were seeded in a density of 31.000 cells/cm² on TCPS and cultured at 37 °C with 5% CO_2_ in a humidified atmosphere for 14 days. The medium was changed three times a week. Cells were quantified with Alizarin red to visualize possible calcium deposits as a result of the osteogenic differentiation.

Alizarin red staining: Staining solution was prepared by dissolving 1.37 g Alizarin red powder (Sigma Aldrich, Germany; 342.3 g/mol) in 100 mL aqua ad iniectabilia (B. Braun, Germany) and filtrated. Cells were fixed for 1 h at room temperature with 70% ethanol and washed three times with aqua ad iniectabilia for five minutes. Samples were stained with alizarin red for 10 min and washed three times with PBS (Gibco, Germany) for 5 min before visualization by light microscopy.

### 4.5. Isolation and Purification of RNA

For RNA isolation, cells in each well were first washed with 2 mL phosphate-buffered saline (PBS; Gibco) and the cells were harvested with 0.5 mL TRIzolTM Reagent (Thermo Fisher Scientific, USA), two wells were pooled. This leads to biological triplicates for each condition. After isolation according to the manufacturers’ instructions, the RNA yield of each sample was verified photometrically at 280 nm and 260 nm (Nanodrop OneTM, Thermo Fisher Scientific, USA). Afterward, RNA purification was performed with Quick-RNA MicroPrep kit (Zymo Research Europe GmbH, Freiburg, Germany) following the producers’ protocol including an on-column DNA digestion. In order to control the success of the purification and to ensure a uniform cDNA synthesis, each sample was measured again (Nanodrop OneTM).

### 4.6. Quantitative Realtime-RT-PCR Analysis (RT-qPCR)

The RNA was transcribed into cDNA (SuperScript III RT, Thermo Fisher Scientific, USA) with a final concentration of 25 ng/µL. All steps from RNA isolation to cDNA synthesis were performed in parallel for all samples of each experiment in order to avoid experimental variations. RT-qPCR was performed in technical duplicates using 2.5 ng/µL cDNA in each reaction and a primer concentration of 0.5 µM. The qTower3 (Analytik Jena, Jena, Germany), High Green Mastermix (Thermo Fisher Scientific, USA), qPCRSoft 3 (Analytik Jena, Germany) and self-designed intron spanning primers were used (Eurofins Genomics, Luxembourg). Primers were designed by using Primer-BLAST (NCBI, Bethesda, MD, USA) followed by a PCR-Check (Eurofins Oligo Analyse Tool, Luxembourg) to ensure in silico PCR specificity. RT-qPCR protocol was performed as follows: 2 min 50 °C for 2 min, 95 °C for 10 min followed by 40 cycles of 95 °C/15 s, 60 °C/30 s and 72 °C/30 s. After 95 °C for 15 s as the last step, a melting curve (60–95 °C). Gene, primer and target/amplicon information for the reference and target genes are displayed in Table 1.

### 4.7. ELISA

To analyze the level of IL-6, a commercially available enzyme-linked immunosorbent assay (ELISA) kit for IL-6 (CSB-E04638h, Cusabio Wuhan Huamei Biotech Co., Wuhan, China) was used following manufacturers’ instructions with fresh cell culture supernatant.

### 4.8. In Vitro Compressive Stimulation Model

The hPDL cells were cultured in high-glucose Dulbecco’s Modified Eagle’s Medium (DMEM; Gibco, USA), containing 100 units/mL of penicillin, 100 g/mL of streptomycin (Gibco, Gaithersburg, MD, USA), 10% FCS (Gibco, USA) and 50 mg/l L-ascorbic acid (Sigma-Aldrich, USA) at humidified 37 °C and 5% CO_2_. Cells were trypsinized and centrifuged at 300× *g* and 90,000 cells were seeded into 6-well plates and cultured for 4 days till 90% cell density was reached. After the incubation of 24 h, a static force of 2 g/cm^2^ (=0.02 N/cm^2^) was applied to the monolayer with sterile round-glass cylinders (34 mm Ø; 18 g), as described and established by Kanzaki et al. [27].

### 4.9. Isolation of Total Protein Respective Cytoplasmic and Nuclear Fractions

After the treatment, cells were analyzed by immunoblotting. Cells were washed and lysed with (100 µL/well) RIPA buffer. Alternatively, to acquire protein fractions from the cytoplasm and nucleus, the NE-PER™ Nuclear and Cytoplasmic Extraction Reagents kit (ne-per TM Thermo Fischer, nuclear and cytoplasmic extraction reagents Thermo Fisher, USA) was used, according to manufacturers´ instructions. The protein amount was quantified by Bradford assay (Bio-Rad, USA) and 25 µg total protein was used for gel electrophoresis, respective 10 µg for fractionated gel electrophoresis.

### 4.10. Immunoblotting Analysis

The protein lysates from hPDL cells were separated by gel electrophoresis (TGX Stain-FreeTM FastCast™, 12%, Bio-Rad, Hercules, CA, USA) and transferred to nitrocellulose membranes (Trans-Blot Turbo ^®^Turbo TM RTA Transfer kit, Nitrocellulose, Bio-Rad, USA). Membranes were blocked for 1 h at RT in 1× Tris-buffered saline containing 0.05% Tween-20 (TBST) supplemented with 5% BSA. Incubation with the primary antibodies occurred overnight at 4 °C and subsequently with the respective secondary antibody for 1 h at RT. Immunoblot was detected by fluorescence, quantified and normalized by means of a ChemiDoc MP Imaging System (Bio-Rad, USA) with Stain-Free technology and Image Lab™ Software (Version6.01 Bio-Rad, Hercules, CA, USA).

### 4.11. NF-kB/DAPI Staining

To investigate the translocation of Nf-kB, cells with and without compressive mechanical stimulation, as well as with HMGB1 and TLR4 AB were fixed with 3.7% formaldehyde suspension (Carl Roth, Karlsruhe, Germany). Afterward, the cells were permeabilized with PBS, supplemented with 0.1% Triton X-100. Subsequently, samples were blocked in PBS containing 1% BSA (Carl Roth, Karlsruhe, Germany), and incubated overnight with NF-kB antibody (#8242, Cell Signaling Technology, USA) followed by ProLong Gold Antifade Mountant with DAPI (Thermo Fisher Scientific, USA), according to the manufacturer’s protocol. Afterward, the monolayer was covered for preservation with coverslips and examined by immunofluorescence imaging microscopy (Observer 7, Zeiss, Germany).

### 4.12. Statistical Analysis

Graphs show mean ± standard deviations (SD). Data were tested for normal distribution by the Shapiro–Wilk test. Afterward, a *t*-test or one-way analysis of variance (ANOVA) followed by Tukeys’ post hoc test was performed in GraphPad Prism (version 9.0; San Diego, CA, USA). A *p*-value < 0.05 was considered statistically significant.

## 5. Conclusions

In this work, it has been shown for the first time that mechanical compression modulates phosphorylation of AKT and MAPKs (phopho-ERK and phospho-p38) via TLR4 in an in vitro model simulating orthodontic tooth movement in human periodontal ligament cells. Furthermore, the inhibition of TLR4 through a TLR4 monoclonal blocking antibody hinders the upregulation of phospho-AKT caused by compressive force to levels comparable to the control. Therefore, it can be assumed that these signaling pathways are modulated not only by mechanotransduction from mechanoreceptors as described in other works but also by TLR4 via transduction of pattern recognition receptors through specific molecular structures on damaged senescent cells during mechanical stress. Additionally, a moderate compressive force is not able to induce the translocation of Nf-kB and phospho-ERK into the nucleus and the signaling of inflammatory cytokine IL-6 may be induced differently. The present findings provide evidence that TLR4 modulating strategies seem to be effective to regulate clinical orthodontic therapy and might have the potential to treat its inflammatory-related side effects such as mechanical or trauma-induced tooth root resorption and periodontal degeneration.

## Figures and Tables

**Table 1:** Primer information: RT-qPCR gene, primer and target/amplicon information for the reference gene RPL22 and investigated target genes. Tm: melting temperature of primer/specific qPCR product (amplicon), %GC: guanine/cytosine content, bp: base pairs.

Gene Symbol	Gene Name (Mus Musculus)	Gene Function	Accession Number (NCBI Gene Bank)	Chromosoma Location (Length)	5′-Forward Primer-3′ (Length/Tm/GC)	5′reverse Primer-3′ (Length/Tm/GC)	Primer Location	Amplicon Length	Amplicon Location (bp of Start/Stop)	Intron-Flanking (Length)	Variants Targeted (Transcript/ Splice)
RPL22	ribosomal protein L22	translation of mRNA in protein	NM_000983	1; 1p36.31 (2061 bp)	tgattgcacccaccctgtag (20 bp/59.67 °C/55%GC)	ggttcccagcttttccgttc (20 bp/59.4 °C/55%GC)	Exon 2/ Exon 3	98	91/188	yes	yes
IL-6	Interleukin 6	important role in bone metabolism; osteoclastogenesis	NM_000600	7; 7p15.3 (1127 bp)	catcctcgacggcatctcag (20 bp/60.32 °C/60%GC)	tcaccaggcaagtctcctca (20 bp/60.47 °C/55%GC)	Exon 2/ Exon 4	164	240/403	yes	yes
IL-8	Interleukin 8	important role in bone metabolism; osteoclastogenesis	NM_000584	4; 4q13.3 (1642 bp)	catactccaaacctttccacc (21 bp/57.9 °C/47,6%GC)	cttcaaaaacttctccacaacc (22 bp/56.9 °C/40.9%GC)	Exon 2/ Exon 3	167	206/372	yes	yes
VEGF A	vascular endothelial growth factor A	induces proliferation and migration of vascular endothelial cells	NM 001171623	6p21.1 (3660 bp)	GGAGGGCAGAATCATCACGAA (21 bp/60.1 °C/52.3%GC)	GGTACTCCTGGAAGATGTCCAC (22 bp/59.8 °C/54.5%GC)	Exon 2/ Exon 3	100	1153/1211	yes	yes
PTGS2 COX2	prostaglandin-endoperoxide synthase 2	involved in prostaglandin synthesis	NM_000963	1q31.1 (4510 pb)	GATGATTGCCCGACTCCCTT (20 bp/59.8 °C/55%GC)	GGCCCTCGCTTATGATCTGT (20 pb/59.6 °C/55%GC)	Exon 4/ Exon 5	185	560/725	yes	yes

## Data Availability

The data that support the findings of this study are available from the corresponding author, R.B.C., upon reasonable request.
